# Supplementary material for: In silico analysis of local RNA secondary structure in influenza virus A, B and C finds evidence of widespread ordered stability but little evidence of significant covariation
Source: Sci Rep. 2022 Jan 10;12:310. doi: 10.1038/s41598-021-03767-x (PMC8748542; doi:10.1038/s41598-021-03767-x)
Supplement: Supplementary file 1 — Supplementary Information. [file 41598_2021_3767_MOESM1_ESM.zip › Supplemental Files/Supplemental File 3.zip!/Supplemental File 3/CM-CC/pdf/ICV_seg6_2_1.fold.R2R.sto.pdf]

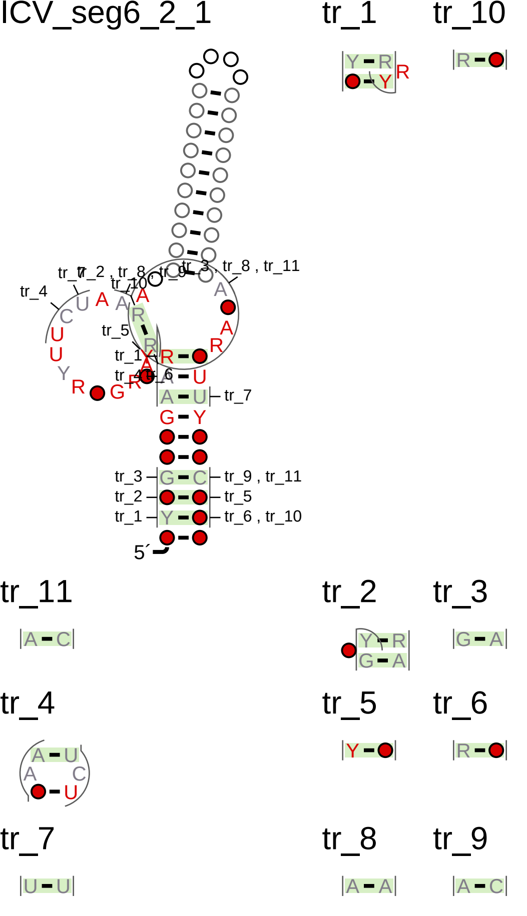
ICV_seg6_2_1
tr_1
tr_10
tr_3 , tr_8 , tr_11
tr_4
tr_5
tr_7
tr_3
tr_2
tr_1
tr_9 , tr_11
tr_5
tr_6 , tr_10
5´
tr_11
tr_2
tr_3
tr_4
tr_5
tr_6
tr_7
tr_8
tr_9
